# Application of a Bayesian nonparametric model to derive toxicity estimates based on the response of Antarctic microbial communities to fuel-contaminated soil

**DOI:** 10.1002/ece3.1493

**Published:** 2015-06-13

**Authors:** Julyan Arbel, Catherine K King, Ben Raymond, Tristrom Winsley, Kerrie L Mengersen

**Affiliations:** 1 Collegio Carlo AlbertoMoncalieri, Italy; 2 Australian Antarctic DivisionKingston, Tasmania, 7050, Australia; 3 Queensland University of TechnologyBrisbane, Queensland, Australia

**Keywords:** Antarctica, dependent models, Fuel spills, Griffiths–Engen–McCloskey distribution, Shannon diversity index, soil biodiversity, species abundance

## Abstract

Ecotoxicology is primarily concerned with predicting the effects of toxic substances on the biological components of the ecosystem. In remote, high latitude environments such as Antarctica, where field work is logistically difficult and expensive, and where access to adequate numbers of soil invertebrates is limited and response times of biota are slow, appropriate modeling tools using microbial community responses can be valuable as an alternative to traditional single-species toxicity tests. In this study, we apply a Bayesian nonparametric model to a soil microbial data set acquired across a hydrocarbon contamination gradient at the site of a fuel spill in Antarctica. We model community change in terms of OTUs (operational taxonomic units) in response to a range of total petroleum hydrocarbon (TPH) concentrations. The Shannon diversity of the microbial community, clustering of OTUs into groups with similar behavior with respect to TPH, and effective concentration values at level *x*, which represent the TPH concentration that causes *x*% change in the community, are presented. This model is broadly applicable to other complex data sets with similar data structure and inferential requirements on the response of communities to environmental parameters and stressors.

## Introduction

An understanding of the environmental processes that affect ecosystems is of fundamental importance for their management and conservation. Ecotoxicology is primarily concerned with predicting the effects of toxic substances on the biological components of the ecosystem. Toxicity data based on the response of a range of native biota are critical to the derivation of site-specific environmental quality guidelines. These include trigger values or contaminant thresholds, which when exceeded prompt remediation and/or cleanup activities. They also include remediation targets which define an acceptable level of ecosystem recovery and restoration and, once reached through remediation, enable sign-off of sites as no longer posing significant environmental risk.

While ecotoxicological assessments aim to predict the effects of contaminants on an ecosystem, monitoring and characterizing the state of an entire ecosystem is rarely practicable. Thus, toxicity tests are generally conducted on single species (populations), or groups of species (communities), as indicators of the overall system state. In remote, high latitude environments such as Antarctica, where field work is logistically difficult and expensive, and where terrestrial ecosystems are comprised of relatively few species and simple food webs, appropriate modeling tools using microbial community responses can be valuable as an alternative to traditional single-species toxicity tests. Such community assessments may provide more representative and relevant information that incorporates complex interactions between species compared with simple single-species tests.

Modeling the responses of species or communities to contamination gradients is conceptually very similar to the broader goal of modeling species responses to environmental conditions, which is an area of long-standing study in the ecological sciences. Conventional observational ecological studies have generally dealt with a relatively small number of species, and the modeling methods have been developed accordingly. Thus, while methods for single-species modeling are relatively mature and diverse (e.g., Elith et al. 2006), community modeling methods are less well established. The development of community modeling methods has, at least in part, been driven by the emergence of high-throughput microbial and similar studies, which can provide information on tens of thousands of species simultaneously, many of which can be extremely sparse. One approach to community modeling is to model single species in an independent fashion and then assemble the individual model predictions into a composite prediction of the community (e.g., Ellis et al. 2011). However, such approaches typically struggle with rare species, which are difficult to model with confidence because of their sparse observations. Appropriate propagation of the uncertainty in individual species models into the composite predictions can also be difficult. An alternative approach, which has become more common in recent years, is to simultaneously model the response of the community as a whole. This can involve the modeling of univariate summaries of multispecies responses, such as compositional dissimilarity (e.g., Ferrier and Guisan 2006; Ferrier et al. 2007) or rank abundance distributions (Foster and Dunstan 2010). Alternatively, the responses of multiple species can be modeled simultaneously (e.g., Foster and Dunstan 2010; Dunstan et al. 2011; Wang et al. 2012).

A common approach to characterizing single-species responses is through the probability of presence 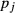 of each species *j* at a site, often as a function of the environmental conditions at that site. This approach can be extended to multiple species using a multiresponse model. The multinomial distribution, which generalizes the binomial distribution to the case where there are more than two species, provides an intuitive framework when the sampling process consists of independent observations of a fixed number of species. This distribution gives the probability of observing any given combination of species, conditional on parameters which are the species relative proportions. This intuitive modeling of species relative proportions has led to the recent popularity of multinomial methods in ecological (e.g., Bohlin et al. 2009; Fordyce et al. 2011; De’ath 2012; Holmes et al. 2012) and genomic (Dunson and Xing 2009) applications. The multinomial approach also provides a natural link to indices that describe various community properties of interest to ecologists, such as species diversity, richness, and evenness. The literature on diversity in ecology is extensive, see, for example, Hill (1973); Patil and Taillie (1982); Foster and Dunstan (2010); Colwell et al. (2012); De’ath (2012). We focus here on the Shannon index, cf Section “Methods”.

In ecotoxicological studies with contaminants, interest is generally directed toward estimating the concentrations of toxicants that cause a certain level of impact on a population or community. The *effective concentration*, or 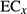 value, is the concentration that causes *x*% effect on the population relative to the controls (e.g., Newman 2012). For example, the 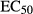 is the median effective concentration and represents the concentration of a toxicant which induces a response halfway between the control baseline and the maximum after a specified exposure time (see section “Methods” for a more detailed definition). Of more ecological relevance in terms of protecting the ecosystem, estimates of lower effective quantiles such as the 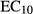 and 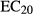 are also commonly estimated. These more sensitive estimates are included in species sensitivity distribution (SSD) models, which are in turn used to derive appropriate protective guidelines on contaminant concentrations. Currently, it is not clear how to best calculate 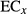 values using whole-community data and to identify the vulnerable members within.

In this study, we apply the modeling method presented by Arbel et al. (2013) to a soil microbial data set acquired across a hydrocarbon contamination gradient at the site of a fuel spill in Antarctica. The method used here extends that presented by Holmes et al. (2012). It allows for an unknown a priori number of species, accounts for additional factors by introducing dependence into the model, and allows predictions to be made at any value of the dependent variable (i.e., contaminant concentration value). This brings estimates which are more efficient, both computationally and inferentially, and allows assessment of the response of species, for instance, in terms of diversity, to contamination. This method provides a robust and practical way to assess a mixed community of organisms (whether microbes or macroorganisms) and determine their responses to a toxicant. There are many studies that focus on the response of a single organism, but often this response will have repercussive effects on a community and the surrounding ecosystem. This method provides a platform by which to explore those effects and derive toxicology data for multiple species without the need for empirical data.

## Methods

### Soil sample collection and analysis

Soil samples were collected from a range of sites across a fuel contamination gradient at Australia’s Casey Station in East Antarctica (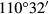
E, 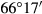
S). The data comprise counts of a large number (of the order of 1800) of microbial taxa, referred to as OTUs (operational taxonomic units; see Schloss et al. 2009), collected at 22 sites, across a range of hydrocarbon contamination (Siciliano et al. 2014). Genomic DNA extracted from samples was sequenced on a 454 Titanium FLX instrument (Roche, Branford, CT) at the Research and Testing facility (Lubbock, TX) using the universal bacterial primers 28F and 519R (Dowd et al. 2008). Pyrosequencing data were processed using the mothur software package (Schloss et al. 2009). This involved removal of short reads (<150 bp), excessive homopolymeric reads (>8 bp repeats) and denoising with AmpliconNoise (min/max flows 360/720) (Quince et al. 2011). Preclustering at 1% was performed to negate the per-base error rate of the 454 platforms. Seed sequences were then aligned to the SILVA 16S rRNA gene database alignment using a NAST alignment algorithm (Pruesse et al. 2007; Caporaso et al. 2010). Reads were then chimera-checked (Edgar et al. 2011) and clustered into OTUs at 96% sequence similarity to achieve approximately species-level units as derived by Kim et al. (2011). Seed sequences from each OTU were then classified using a Naïve Bayesian classifier in mothur against the Greengenes 16S reference database (October 2012 version, see McDonald et al. 2012).

While a range of geographic, environmental, and soil physicochemical measurements were taken as covariate data for each sample, in this study we specifically focus on the concentration of fuel in the soil, measured as total petroleum hydrocarbon (TPH) in mg/kg of soil. TPH concentrations in each soil sample were measured using a gas chromatograph with flame ionization detection (GC-FID) via hexane extraction, as described by Schafer et al. (2007). Total signal in the C9–C28 range was measured to determine TPH concentrations.

Although a continuous variable, the same TPH concentration was recorded for several sites, ranging from 0 to 22,000 mg TPH/kg soil. This is the case for the baseline which comprises 10 uncontaminated sites (i.e., with zero TPH). The statistical model expresses the count of each OTU as a function of the environmental covariates. In order to accommodate for multiple sites with identical TPH concentrations, the ties in concentration were jittered, that is, had a random Gaussian noise added (absolute value for the case TPH = 0). This noise can be interpreted as errors in the measurement process and be incorporated in the probabilistic model. Reproducing the estimation for different small values of variance for that noise compared with the variability in observed TPH, we have noted that the results were not substantially altered.

For computational reasons, only the most abundant OTUs were included in the analyses. Here, only OTUs with total abundance exceeding 10 over all sites were included, which occurred for 392 OTUs. However, we show in the Appendix that repeating the analyses by including or discarding up to 20% of OTUs did not substantially alter the results.

### Statistical model

A brief summary of the modeling procedure is given here: Full details of the model are provided by Arbel et al. (2013). The following notation is used to refer to the data. Each unique covariate value, possibly after jittering, is indexed by *i* and is denoted by 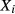, *i* = 1,…,*I*. Recall that this may correspond to a single site or to a collection of sites with the same covariate value. However, for the sake of simplicity, we refer to *i* as the site index (i.e., “site *i*”). OTUs are indexed by *j* = 1,2,…, so that the abundance of OTU *j* at site *i* is denoted by 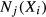 and the total abundance of all OTUs at site *i* by 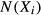. The same notation, using *p* instead of *N*, relates to proportions, or average proportions, rather than abundances. That is, 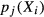 is the probability of observing OTU *j* at site *i*. The probability distribution 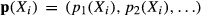, denoted with a bold letter for a vector, is the distribution of these probabilities across all OTUs at site *i*, that is, the community composition. The dependence of the community composition on the covariate *X* is modeled by:

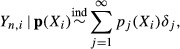
where the *n*th observation made at site *i* is denoted by 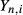, for 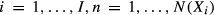, and 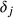 stands for taxa *j*. That is, observation is OTU *j* with probability 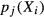.

Not all taxa in the population are assumed to have been observed, and no a priori value is placed on this total number, which might be much larger than the number of observed taxa. Note that we do not attempt to estimate the number of undetected taxa (see e.g., Royle and Dorazio 2008 for an extensive account on the role of imperfect detectability).

A Bayesian perspective is adopted for estimation of this model. The basic machinery requires specification of the prior distributions of the community vectors 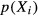. It takes the form of a probability distribution, which represents a reasonable prior knowledge about the parameters of the model. It is updated with the data and the sampling model through the celebrated Bayes’ rule in order to obtain the posterior distribution of the parameters. All inferential information is enclosed in that distribution. A common approach to estimating such models is through Markov chain Monte Carlo methods (MCMC), which (approximately) sample from the posterior distribution. Hence, the inference from the fitted model makes use of a so-called posterior sample, which is a random sample of probabilities 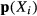 drawn from the posterior distribution.

A common prior distribution for models of this type is the Griffiths–Engen–McCloskey distribution, denoted by 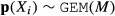, and defined by

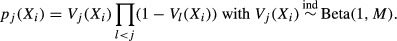


This construction of generic probability weights 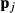 is often described using the analogy of breaking a stick. Start with a stick of length 1, break it at a random length 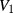, and define 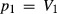. Do the same for the remaining stick of size : break it at a random length 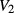, define 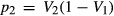, and continue iterating with this process. The remaining length at stage *j*, 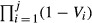, goes to zero when *j* goes to infinity, and so, the 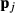
’s sum to 1. Commonly, independent priors on 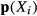 are used for different 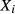 (e.g., Holmes et al. 2012). However, as we are dealing with a continuous covariate, it is arguably more appropriate to adopt a dependent prior that evolves smoothly with the covariate *X*: a small change in 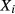 induces a small change in 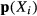. The GEM distribution can thus be extended to a dependent version with this smoothness constraint, denoted here by DepGEM. Both models will be compared in our analyses.

The GEM prior on the vector 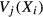 is required to be marginally beta-distributed. The DepGEM prior additionally requires that 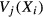 exhibit dependence across *i* in order to be smooth with respect to the covariate *X*. This is achieved using Gaussian processes 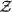, whose covariance matrices allow a very flexible modeling of the dependence through a so-called *bandwidth* parameter. Informally speaking, the bandwidth can be thought of as roughly the distance one has to move in the covariate space before the process can change substantially. Also, the model allows estimation of the probability 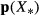 for covariate values 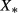 that are unobserved, by computing the predictive distribution of the Gaussian processes. See Arbel et al. (2013) for details.

**Figure 1:**
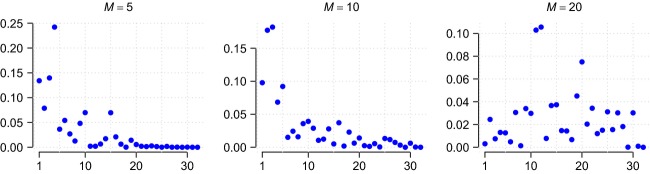
Proportions 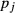 sampled from the Griffiths–Engen–McCloskey distribution. *From left to right*: precision parameter *M* = 5,10,20 (different *y*-axis scaling). The *x*-axis represents the species index *j* = 1,2,…. The diversity of the sample increases as the precision parameter *M* increases (left to right). Large values of *M* tend to give more uniformly distributed probability values.

The parameter *M* is called the *precision parameter* of the prior and governs the uniformity of the probabilities drawn from it. Higher values of *M* yield a more uniform probability distribution, which is equivalent to a more diverse community, and so, *M* effectively controls the level of diversity in the prior. For small *M*, the first few taxa share most of the weights, whereas in the limiting case *M*, the weights tend to be uniformly distributed, cf Fig. 1. Exploratory analyses indicate that, given a sufficiently large range of *M*, the OTU frequencies drawn from this distribution are similar to those observed in the data. A random prior distribution on *M* can be used to ensure that it has adequate range.

A means of studying communities diversity is through the *Shannon diversity index* (also known as the Shannon–Wiener index, the Shannon–Weaver index, and the Shannon entropy), defined by

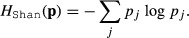
3

**Figure 2:**
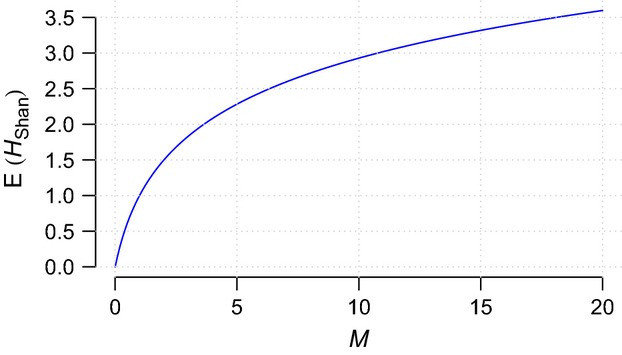
Prior expectation of the Shannon diversity index under GEM distribution. The *x*-axis represents the precision parameter *M*.

The prior expectation of the Shannon index under the GEM prior is given by

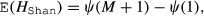
where *ψ* is the *digamma* function (see Cerquetti 2014). Figure2 illustrates the a priori effect of *M* on the Shannon index.

The estimation of the model is described in Arbel et al. (2013). Posterior sampling is performed by a Gibbs algorithm, with a Metropolis–Hastings step for nonconjugate conditionals. It is run for 50 000 iterations thinned by a factor of 5 with a burn-in of 10 000 iterations. The parameters of the hyperpriors are chosen so that they are weakly informative. The efficiency and convergence of the sampler was assessed by trace plots and autocorrelations of the parameters, which did not reveal any convergence issues.

### Inference from fitted models

Fitted models can be used to make a range of inferences about the microbial taxa and the response of the community to contamination.

**Figure 3:**
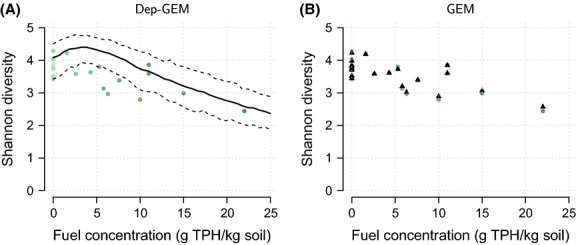
Diversity estimation results. (A) DepGEM model estimates (50,000 MCMC samples). Thick line: Shannon diversity estimate. Dashed lines: credible interval for the diversity index. Dots: Shannon diversity index in raw data. (B) GEM model estimates (50,000 MCMC samples). Triangles: posterior mean estimate of the Shannon diversity index.

Diversity, defined in equation 3 by the Shannon index as noted above, was estimated from the posterior sample obtained from the Gibbs algorithm. It consists of a sample of probabilities **p**(*X*) (i.e., estimates of community composition). The Shannon index can be calculated for each of these samples, yielding a posterior estimate of the Shannon index, which is illustrated in Fig. 3, for both GEM and DepGEM models.

On the basis of their fitted responses, OTUs were classified as increasing or decreasing with contamination. The classification was based on the difference 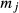, for each OTU *j*, between its average probability at low contaminant (i.e., 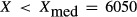 mg/kg TPH, the median value of observed TPH) and its average probability at high contaminant (i.e., 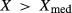). An intuitive interpretation of 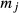 is that it indicates to what extent OTU *j* is impacted by the contaminant. The posterior distribution is available for 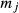, along with 95% credible intervals. The clustering was conducted as follows: if zero was on the left of the credible interval (i.e., 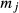 was deemed from the posterior distribution to be highly likely to be larger than zero), then OTU *j* was classified as decreasing with TPH; if zero was on the right, it was classified as increasing. Otherwise (if the credible interval included zero), it was deemed that the model did not give enough information about the response of OTU *j* to the contaminant, and it was given a “no classification” label. The classification was performed for both the GEM and the DepGEM models. We also classified each OTU directly using the raw data, from which no credible interval is available, and so, no OTUs were placed in the “no classification” group in this case.

In order to obtain an overview of the ecological relevance of these classification results, we compared the results to a list of 181 genera of known hydrocarbon-degrading bacteria (Prince et al. 2010). This list is based on published results from a wide range of ecosystem types – not just Antarctic soils – and so, this comparison was only expected to give general indications of ecological relevance. We expected that genera known to be hydrocarbon degraders would typically show an increasing response to contamination, whereas the reverse would be true for other genera. The sensitivity (true positive rate) and specificity (true negative rate) were calculated on the basis of the number of correctly classified OTUs (excluding those OTUs that were given “no classification” by the model). The weighted sensitivity and specificity were also calculated by taking into account the relative abundances of the OTUs. Note that these comparisons were only conducted using those OTUs identified to a taxonomic level of genus or finer.

The 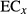 value (see e.g., Newman 2012) is *the TPH concentration associated with an x*% *response in the target organisms*. For single-species studies, this is commonly assessed by an *x*% increase in mortality or some sublethal response and is determined using logistic or probit regression. In applications with a multispecies response, it is the response of the community as a whole that is of interest. This can be defined in a number of ways depending on the specific aspects of interest to the ecological application. In an application where the Shannon index is deemed to be an appropriate community indicator, the estimated Shannon index curve (e.g., Fig. 3) could be used to define the 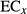 threshold. As an alternative, we illustrate the use of a dissimilarity index as a measure of change in community composition. Many dissimilarity functions are available; for the purposes of demonstration, we adopted the commonly used Bray–Curtis dissimilarity (Bray and Curtis 1957). We defined the baseline community as the ten uncontaminated sites, where TPH equals zero. To calculate an 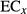 value, we seek the TPH level that corresponds to an *x*% change in the community composition relative to the baseline. The dissimilarity at TPH zero (labeled 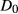 here) is an estimate of the variability in community composition between uncontaminated sites and so is greater than zero. The Bray–Curtis dissimilarity has a maximum value of 1, which occurs when two sites have no species in common (i.e., disjoint community compositions). For 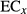, we therefore calculated the 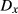 threshold as 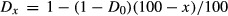. This is equivalent to assuming that an 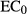 value (i.e., no change relative to baseline) occurs at 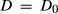, an 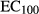 value (i.e., 100% change in composition) occurs at *D* = 1, and with linear interpolation for intermediate values. The dissimilarity curve is not guaranteed to be monotonic, and so, there may be several TPH values associated with a certain dissimilarity level 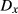. In this situation, the smaller value should generally be used, so as to provide a conservative 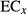 estimate. It is unlikely that this dissimilarity threshold will coincide exactly with one of the measured TPH levels in the data, and so, this approach required the model to be able to interpolate between observed TPH values. This is one of the features of the DepGEM model. This approach also allowed the uncertainty in the 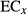 value to be estimated, by considering the 95% credible intervals on the estimated dissimilarity. As the curves of the credible intervals are similarly not necessarily monotonically increasing, it may be necessary to define an increasing envelope of them in order to derive the credible intervals for 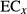 values. Note that this technique leads to conservative estimates for the uncertainty about 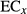 values, as it enlarges the proper credible intervals for the dissimilarity.

### Results for Microbial Data

Estimates of diversity with respect to hydrocarbon contamination are shown in Fig. 3. The DepGEM model (Fig. 3A) suggested that diversity first increases with TPH with a maximum at 3000 mg TPH/kg soil and then decreases with TPH. The GEM model estimates are shown for comparison in Fig. 3B. These estimates showed more variability with respect to TPH, being closer to the estimates of the diversity from raw data. Note that the GEM estimates were only available at levels of the covariate that were present in the data, because of the independent nature of the model specification. The DepGEM, in contrast, provided predictions across the full range of TPH values.

The clustering results are summarized in Table1. Our data included 64 distinct bacterial genera, of which 22 also appeared on the list of known hydrocarbon-degrading taxa. Table1 shows the comparison of the classification results to the list of known hydrocarbon-degrading genera. Broadly, the results of all methods showed a reasonable match to the expected responses. The GEM model gave more “no classification” results than the DepGEM model. This illustrates the fact that the GEM model uses less information than the DepGEM (which, for each contaminant value, borrows information to neighboring contaminant values for the estimation), hence has larger credible intervals which tend to include zero more often. This means that, although the sensitivity and specificity of the GEM model were similar to that of the DepGEM, more OTUs were left unclassified by the GEM approach compared to the DepGEM. The sensitivity and specificity of all methods were generally higher when weighted by OTU abundance (rather than simply calculated on the number of OTUs correctly classified). This suggests that high-abundance OTUs were typically deemed to have a response that matched the expected behavior. This may be because the higher-abundance OTUs are better modeled by these methods, and so, their response is more likely to be correctly characterized. Alternatively, low-abundance OTUs may simply not express the response type that might be expected for their genus. OTUs from a hydrocarbon-degrading genus, if present only in low numbers, might not tend to increase in response to the presence of hydrocarbons because they are outcompeted by other, more abundant hydrocarbon-degrading bacteria. All methods showed generally low values of sensitivity (i.e., not all OTUs from hydrocarbon-degrading genera actually showed increasing responses to contamination). This is consistent with the nature of the list of hydrocarbon-degrading bacteria, which was drawn from a diverse range of studies covering many ecosystem types. Particular species found to be associated with hydrocarbon degradation in those studies might not match the species within the same genera found in our samples. Additionally, the model was estimated separately on the three groups. We plot the results for diversity and dissimilarity estimation in 5Fig. in Appendix A. Note that the decreasing/increasing pattern of the groups with respect to TPH is not visible in the graphs as it is defined in terms of probability of the species, while we plot diversity and dissimilarity.

**Table 1:** Comparison of the clustering to taxonomic information. Clustering is performed according to the models (Data: raw data, DepGEM: dependent model, GEM: independent model)

	Sensitivity (Abundance-weighted sensitivity)	Specificity (Abundance-weighted specificity)
DepGEM	0.35 (0.62)	0.91 (0.93)
GEM	0.41 (0.63)	0.80 (0.90)
Data	0.43 (0.63)	0.72 (0.81)

	Known hydrocarbon-degrading genera			Other genera			
	N OTUs classified as increasing	N OTUs classified as decreasing	N OTUs not classified (no classif. rate)	N OTUs classified as increasing	N OTUs Classified as decreasing	N OTUs not classified (no classif. rate)
DepGEM	18	34	13 (0.20)	6	58	18 (0.22)
GEM	15	21	29 (0.45)	6	24	52 (0.63)
Data	28	37	–	23	59	–

**Figure 4:**
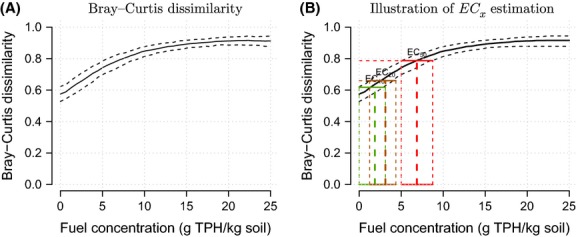
Dissimilarity and 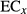 estimation results. (A) Posterior distribution (DepGEM model) of the dissimilarity between the control community (TPH equals zero) and other sites (color dots show the TPH levels actually present in the data). The thick line shows the mean estimate, including TPH values in between those actually observed. The dashed lines give 95% credible intervals of the dissimilarity estimate. (B) Illustration of estimation of 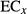 values and credible intervals.

The estimated compositional dissimilarity curve with TPH is shown in Fig. 4, along with the 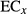 values extracted from it and provided in Table2. Dissimilarity generally increased with TPH, illustrating that the contaminant alters community structure. Typically, 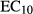, 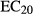 and 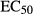 values, cf Table2, are reported in toxicity studies to be used in the derivation of protective concentrations in environmental guidelines or for comparisons of sensitivity between biota. 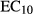, 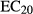, and 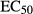 values estimated from this model are 1875, 3125, and 6875 mg TPH/kg soil, respectively. For small *x* (<10%), the lower bound of the credible interval on the 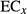 value is zero, because both TPH and dissimilarity values are bounded below by zero. Conversely, for large *x* (more than 75%), the upper bound on the credible interval is 25,000, which is the limit of the TPH range in our analysis.

**Table 2:** Estimates of mean 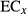 values and their 95% credible intervals for fuel based on microbial community dissimilarity (all units are mg TPH/kg of soil)

X	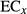	min	max
5	1250	0	2500
10	1875	0	3125
15	2500	1250	3750
20	3125	1250	4375
25	3125	1875	4375
30	3750	2500	5625
35	4375	3125	6250
40	5000	3750	6875
45	6250	4375	8125
50	6875	5000	8750
55	8125	6250	10,000
60	9375	6875	11,250
65	10,625	8750	13,125
70	12,500	10,000	16,250
75	15,000	11,875	25,000
80	20,625	14,375	25,000

As a sensitivity analysis of the performance of the model, we have estimated the model on modified data, by

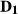
 Deleting the least abundant taxa (fixing the threshold total abundance limit at 12 instead of 10 in the default data set), which amounts to a decrease of 15% in the number of taxa,

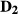
 Including additional taxa up to total abundance of 8 (instead of 10 in the default data set), which amounts to an increase of 19% in the number of taxa,

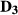
 Excluding randomly 5 sites of 22 in the default data set, that is, 23% of the sites.

As the plots of the results in 6Fig. in Appendix B show, the model is fairly robust to these modifications of the data as the estimations are consistent with Figs. 3 and 4.

## Discussion

In this study, we applied a dependent Bayesian model to soil microbial data, to assess the effects of hydrocarbon contamination on microbial communities. While many ecotoxicology studies are conducted using single species, in microbial analysis, whole communities are generally considered. Some studies have considered the effects of toxicants on microbial communities, but with the advent of next-generation sequencing technologies, we are now able to accurately identify members of the communities to make reliable inferences (Vazquez et al. 2009). Our method allows community-level analyses to derive toxicology end points and to understand the effects of contaminants closer to the whole-of-ecosystem level. This is novel in the field of microbiology and can provide insight to the harmful effects of pollutants, because microbes are typically the most sensitive members of the ecosystem and can therefore potentially be used as an indicator of contamination impacts (van Dorst et al. 2014). While statistical methods to determine point estimates for single-species tests are well established (e.g., probit analysis, trimmed Spearman Karber), standard methods to determine the sensitivity of whole communities and to derive single point estimate values are currently lacking. In addition, the responses of whole communities to stressors are often poorly understood. Such community-based assessments are becoming more important, especially in harsh environments such as Antarctica in which plant and invertebrate communities typically display low diversity, and are difficult to measure responses in. The method presented here has potential value in other applications that consider changes in community composition with respect to environmental or other processes. More broadly, the method can be applied to any set of categorical response variables indexed by integers. As seen in the introduction, the literature on diversity in ecology is extensive, but we note that similar indices arise in other areas of science, such as biology, engineering, physics, chemistry, economics, health, and medicine (see Havrda and Charvát 1967; Borges and Roditi 1998; Kaniadakis et al. 2005) and in more mathematical fields, such as probability theory (Donnelly and Grimmett 1993), which are possible fields of application of our methodology.

There are several computational limitations of the approach: The model is difficult to estimate on very large data sets, even if extremely sparse as is the case of the original ecotoxicological data set studied here. Indeed, the sparsity of the data is a source of computational difficulty with the posterior computation (see Arbel et al. 2013). However, in terms of diversity, most of the information is driven by the largest OTUs, and so, working on a subsample of the data is satisfactory in this respect. We used only the most abundant OTUs, but showed that the results were not sensitive to the abundance threshold used for inclusion.

An attractive feature of the dependent model is that it allows predictions at arbitrary covariate levels, not just those values that were present in the data. This allows inferences to be made across the full range of covariate values (and, with care, possibly beyond the range of covariate values actually sampled). This is a particularly useful consideration in several situations, for instance, where experimental data are sparse, such as in polar applications that involve costly and logistically difficult fieldwork, or in situations in which a critical contaminant level lies in between two tested TPH levels (as with the calculation of 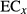 values demonstrated here). However, extra care should be taken with the interpretation of estimates that lie between sampled values as they are typically driven by the assumptions on the model (e.g., smoothness constraints) rather than by the data.

In Holmes et al. (2012), the total number of species is assumed to be known. In this case, the observational model is equivalent to the multinomial model at the species level. This assumption is a limitation of that model specification, in the sense that incorrect assumptions for the total number of species might alter the resultant estimations. This limitation is overcome by the nonparametric structure of the DepGEM model.

There are many ways in which this work can be extended. Additional factors to TPH, which include other types of environmental factors about soil composition, geographic factors, etc, could be utilized in the model. This extension to multiple covariates would be more demanding to fit numerically, although with little additional coding cost. This would allow interactions between predictor variables to be specified a priori by the covariance structure of the Gaussian processes. The model could also handle categorical covariates (as well as a mix of continuous and categorical) using a latent continuous variable (say a normal) for each of them and then using, for example, its sign in the case of a binary variable.

This study brings some new elements to the understanding of the effects of contaminants on ecosystems. The grouping is a valuable information for knowing which OTUs are to be studied for deriving environmental quality guidelines, while the 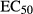 and 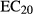 estimates allow fixing thresholds in these guidelines.

The sensitivity and specificity of all methods were generally higher when weighted by OTU abundance (rather than simply calculated on the number of OTUs correctly classified). This suggests that high-abundance OTUs were typically deemed to have a response that matched the expected behavior. This may be because the higher-abundance OTUs are better modeled by these methods, and so, their response is more likely to be correctly characterized. Alternatively, low-abundance OTUs may simply not express the response type that might be expected for their genus. OTUs from a hydrocarbon-degrading genus, if present only in low numbers, might not tend to increase in response to the presence of hydrocarbons because they are outcompeted by other, more abundant hydrocarbon-degrading bacteria.

All methods showed generally low values of sensitivity (i.e., not all OTUs from hydrocarbon-degrading genera actually showed increasing responses to contamination). This is consistent with the nature of the list of hydrocarbon-degrading bacteria, which was drawn from a diverse range of studies covering many ecosystem types. Particular species found to be associated with hydrocarbon degradation in those studies might not match the species within the same genera found in our samples.

Several OTUs were consistently classified as increasing with hydrocarbon contamination, yet did not appear on the list of known hydrocarbon-degrading genera. These were from the genera *Methyloversatilis* (order *Rhodocyclales*), *Propionicimonas* (order *Actinomycetales*), and *Simplicispira* (order *Burkholderiales*). However, other species from these genera are known hydrocarbon degraders (Prince et al. 2010). It is thus possible that the OTUs in our study observed to increase with TPH also possess the genes for metabolic utilization of the various compounds in the fuel (Cooksey et al. 1990; Dressler et al. 1991; Cho et al. 1998).

Diversity of microbial communities, although not strongly affected, was reduced in response to fuel contamination. The 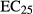 value of 3125 mg TPH/kg soil estimated in this study indicates that relatively small concentrations of hydrocarbons can elicit an effect on the microbial community. Few other point estimates of fuel toxicity are available for Antarctic soil biota. Our results here for microbial diversity are higher than those of Schafer et al. (2009) who reported 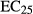 values for microbial community composition and microbial biomass of 800 and 2400 mg TPH/kg soil, respectively. Harvey et al. (2012), using nitrification rates as a surrogate for microbial activity, reported 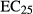 values of 200 and 400 mg/kg for potential nitrification activity and gross nitrification, respectively. However, all of these microbial end points (including the results from the present study) are lower than the findings of Nydahl (2013) who investigated the response of several Antarctic moss species and a terrestrial alga from Casey Station to soil artificially contaminated with fuel. All species were relatively insensitive to fuel contamination with reduced photosynthetic efficiency observed at concentrations ≥25,500 mg TPH/kg soil. *Inhibitory concentrations* (*IC*) were calculated, and 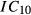 and 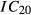 estimates ranged from 21,300 to 61,500 mg TPH/kg soil. Hence, microbial processes and community structure and function appear to be more sensitive indicators of fuel contamination than are the responses of plants in single-species tests. Further work is required using other Antarctic biota including plants and microinvertebrates to assess whether this truly represents an increased sensitivity of microbial species compared to higher taxa.

Degradation of the hydrocarbon present in the soils and survivability of bacteria is a possible explanation for the community profile shifts observed in the data that were examined. However, there are a number of other reasons why the microbes may respond to an exogenous toxicant. Microbial communities are complex and many dependencies and competitive relationships exist (Hosni et al. 2011; Stewart 2012). The restriction of the dominance of abundant taxa can significantly alter the impact on other bacteria. For example, if a dominant species is significantly impacted by the presence of a toxic hydrocarbon, then other species will have the opportunity to thrive and increase abundance, provided they are tolerant of or resistant to the compound. Additionally, the removal of a bacterial species from a community may limit the quantity of secondary metabolites available to a dependent symbiont species that subsequently diminishes in abundance due to this indirect effect of the toxicant (Epstein 2009; Lewis et al. 2010). These effects can at first have a positive impact of community measures such as species richness at low TPH levels, but will always impact negatively as levels rise.

The list of known hydrocarbon-degrading bacteria used here is certainly incomplete. The vast majority of bacteria are unable to be cultured in vitro (Ferrari et al. 2005). In addition to the well-characterized species that degrade hydrocarbons, there are a large range of taxa from extreme environments, such as Antarctica, that are yet to be cultured (Lee et al. 2012). It is highly likely that, given the range of compounds in TPH and the richness of species in the samples examined, there are many more species capable of degradation. Here, the analysis was limited to those taxa that were able to be classified to genus level. However, it is common knowledge that bacterial taxonomy is far behind the rest of the field and many thousands of taxa isolated through molecular surveys remain unclassified (McDonald et al. 2012; Werner et al. 2012; Winsley et al. 2012). If a taxonomy was provided for all OTUs, it is likely that the confidence in this method will increase further as more taxa can be analyzed, making the testing more robust.

Overall, this study presents a novel modeling method for deriving point estimate concentrations in toxicological studies. It is unique in its approach and its ability to work with multiple species and large data sets to produce toxicity estimates which reduce complex multispecies responses to a single sensitivity value that represents the response of the community. The model is broadly applicable to other complex data sets with similar data structure and inferential requirements on the response of communities to environmental parameters and stressors.

## Figures and Tables

**Figure 5:**
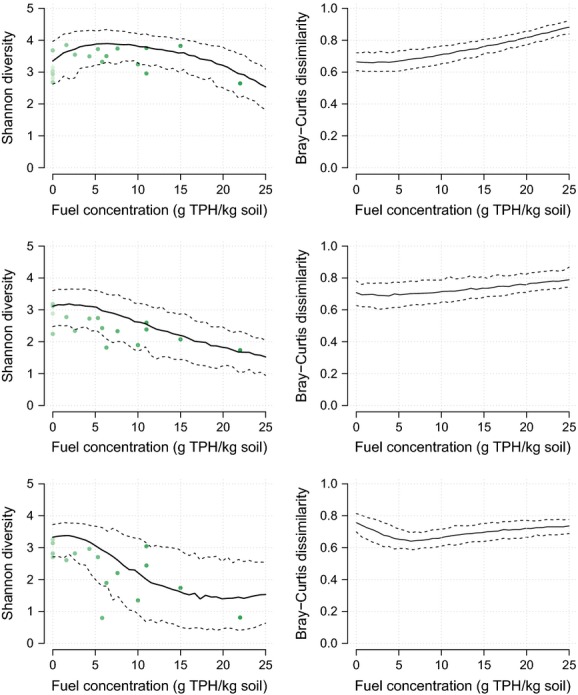
Estimation of the DepGEM model on the three response groups (50,000 MCMC samples). (Left) Diversity estimation results. Thick line: Shannon diversity estimate. Dashed lines: credible interval for the diversity index. Dots: Shannon diversity index in raw data. (Right) Bray–Curtis dissimilarity index and credible intervals. (Top) probability decreasing with TPH, (Middle) probability increasing with TPH, (Bottom) group with no clear pattern (see Section “Results for Microbial Data”).

**Figure 6:**
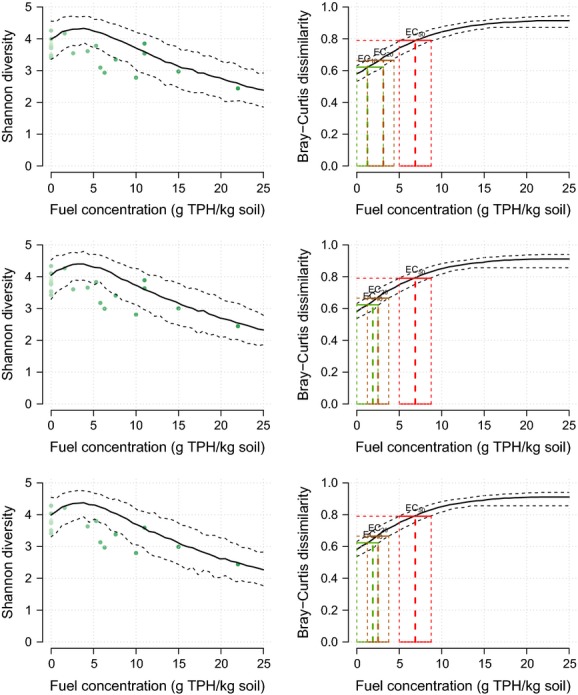
Sensitivity analysis for DepGEM model estimates (50,000 MCMC samples). (Left) Diversity estimation results. Thick line: Shannon diversity estimate. Dashed lines: credible interval for the diversity index. Dots: Shannon diversity index in raw data. (Right) Illustration of estimation of 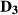 values and credible intervals based on the Bray–Curtis dissimilarity index. (Top) data 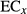, (Middle) data 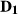, (Bottom) data 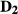 (see Section “Results for Microbial Data”).
